# Correction: Health conditions associated with overweight in climacteric women

**DOI:** 10.1371/journal.pone.0228210

**Published:** 2020-01-16

**Authors:** Maria Suzana Marques, Ronilson Ferreira Freitas, Daniela Araújo Veloso Popoff, Fernanda Piana Santos Lima de Oliveira, Maria Helena Rodrigues Moreira, Andreia Maria Araújo Drummond, Dorothéa Schmidt França, Luís Antônio Nogueira dos Santos, Marcelo Eustáquio de Siqueira e Rocha, João Pedro Brant Rocha, Maria Clara Brant Rocha, Maria Fernanda Santos Figueiredo Brito, Antônio Prates Caldeira, Fabiana Aparecida Maia Borborema, Viviane Maia Santos, Josiane Santos Brant Rocha

The fourteenth and fifteenth authors’ names are spelled incorrectly. The correct names are: Fabiana Aparecida Maia Borborema, Viviane Maia Santos.
