# Supplementary material for: Health conditions associated with overweight in climacteric women
Source: PLoS One. 2019 Dec 12;14(12):e0218497. doi: 10.1371/journal.pone.0218497 (PMC6907811; doi:10.1371/journal.pone.0218497)
Supplement: Attachment — Submitted filename: Response to Reviewers.docx [file pone.0218497.s002.docx]

REBUTTAL LETTER

**Journal Requirements**

1. When submitting your revision, we need you to address these additional requirements.

RESPONSE: Thank you for your observation. We have reviewed and sought to meet PLOS ONE's style requirements.

2. We suggest you thoroughly copyedit your manuscript for language usage, spelling, and grammar. If you do not know anyone who can help you do this, you may wish to consider employing a professional scientific editing service.

RESPONSE: We apologize for the English language errors. This issue has been solved since all the text was submitted for editing by the AJE team.

3. Please provide the full name of the ethics committee which approved this study in the ethics statement in your manuscript and the online submission form.

RESPONSE: Thank you for your observation. The full name of the ethics committee that approved this study has been provided in the ethics statement in our manuscript (Line 181-182).

4. Please cite and discuss the following related work: https://journals.plos.org/plosone/article?id=10.1371/journal.pone.0211617

RESPONSE: Thank you for your observation. The work was cited (Line 58).

The present work is not a replication of prior research but research that is part of a project called “Health problems of climacteric women: an epidemiological study”, which was developed by a research group linked to a postgraduate programme. Strictly speaking, the central theme is the health of climacteric women. This group consists of several researchers who each dealing with one of the following lines of research: metabolic syndrome, mental health, obesity, quality of life, sleep disorders, health perception, urinary incontinence, climacteric symptoms, and levels of physical activity. Thus, the work now presented is a product of the obesity in climacteric women line of research (Line 75-82).

5. Please remove your figures from within your manuscript file, leaving only the individual TIFF/EPS image files, uploaded separately.  These will be automatically included in the reviewers’ PDF.

RESPONSE: Thank you for your observation. The figure have been removed from the manuscript file.

6. ** Please include your tables as part of your main manuscript and remove the individual files **. Please note that supplementary tables (should remain/ be uploaded) as separate "supporting information" files

RESPONSE: Thank you for your observation. The tables have been included as part of the main manuscript and removed from their individual files.

**Reviewer #1**

Comment 1

*“It is necessary that for authors to find someone who is familiar with English to revise this paper”.*

RESPONSE: We apologize for the English language errors. This issue has been solved since all the text was submitted for editing by the AJE team.

Comment 2

*“The authors seemed to be unclear about the independent variables and outcome variables; we can find this confused expression in both of abstracts and the main paper.*

RESPONSE: The suggestion was followed, and the text was changed. The outcome variable is overweight/obesity (Line 35/110-111). Thank you for the suggestion.

Comment 3

*“Regression models are common used in study these years, authors do need to explain their analysis step by step, and the tables are substandard.”*

RESPONSE: The work was performed following the methodological rigor for random sampling, and a considerable number of participants were included. The data collectors were calibrated, and prior to collection, a pilot study was performed. The statistical analysis was conducted appropriately, and the tables were checked by the authors. Thank you for the suggestion (Line 94-97/102-107/ 311-313).

Comment 4

*“Some part of discussion is irrelevant with the results”:*

RESPONSE: The discussion was reviewed by the authors, including suggestions made by the reviewers. Thank you for the suggestion (Line 249-250/252-254/261-265).

**Reviewer #2**

Comment 1

“*please provide the list of abbreviations.*

RESPONSE: The suggestion was followed, and the text was changed. All the meanings of the acronyms have been contemplated in the text. Thank you for the suggestion.

| AMI | acute myocardial infarction |
| --- | --- |
| BDI | *Beck Depression Inventory* |
| BMI | body mass index |
| CAD | coronary artery disease |
| CI | confidence interval |
| CKD | chronic renal disease |
| DEXA | dual X-ray absorptiometry |
| FHS | Family Health Strategy |
| FSH | follicle stimulating hormone |
| HDL | high-density lipoprotein |
| ICIQ-SF™ | International Consultation on Incontinence Questionnaire - Short Form |
| IPAQ | International Physical Activity Questionnaire |
| LDL | low-density lipoprotein |
| MG | Minas Gerais |
| MS | metabolic syndrome |
| NCEP/ATP-III | Third Report of the National Cholesterol Education Program Expert Panel on Detection, Evaluation, and Treatment of High Blood Cholesterol in Adults |
| NHA | National Health Agency |
| NHC | National Health Council |
| PHC | Primary Health Care |
| PR | prevalence ratios |
| S | stature |
| SAH | systemic arterial hypertension |
| SOBRAC | Brazilian Climacteric Society |
| SPSS | Statistical Package for the Social Sciences |
| VLDL | very low-density lipoprotein |
| W | weight |

Comment 2

*“did you perform body composition analisis?”*

RESPONSE: The study used BMI as a diagnostic criterion of overweight/obesity as opposed to using other gold standard techniques, such as X-ray double emission densitometry (DEXA), which allows the analysis of body composition. However, it should be noted that although the use of BMI is a limiting factor, its use is supported by the acceptance of BMI as a diagnostic criterion for obesity by the World Health Organization (Line 311-323).

Comment 3

*“Introduction and Discussion section needs improvement- please cite: doi: 10.1039/C9FO01006H; 10.20452/pamw.4426.; 10.1097/MD.0000000000014909.”*

RESPONSE: These have been cited. Thank you for the suggestion (Line 249-250/252-254/ 261-265).

Comment 4

“*In discussion please provide “study strong points” and “study limitation” section.*

RESPONSE: This was a randomized, probabilistic sample of a representative size, with methodological rigor, data collector training and a pilot study. All of the study procedures were conducted according to current legislation in relation to research ethics. Study limitation: This was a prevalence study that does not allow the establishment of cause-effect conclusions, and there was no use of DEXA for body analysis (Line 311-323/ 330-332).
